# PROTOCOL: Police programs that seek to increase community connectedness for reducing violent extremism behaviour, attitudes and beliefs

**DOI:** 10.1002/cl2.1076

**Published:** 2020-02-25

**Authors:** Lorraine Mazerolle, Adrian Cherney, Elizabeth Eggins, Angela Higginson, Lorelei Hine, Emma Belton

**Affiliations:** ^1^ School of Social Science, University of Queensland St Lucia Campus St Lucia Queensland Australia; ^2^ School of Justice, Queensland University of Technology Gardens Point Campus Brisbane Queensland Australia; ^3^ The Australian Research Council Centre of Excellence for Children and Families Over the Life Course St Lucia Queensland Australia

## Abstract

Community engagement and connectedness are identified as potential mitigating factors for those at risk of engaging in violent extremism. Police have a critical role in promoting social inclusion and social connectedness and thereby preventing violent extremism. Thus, it is essential to understand the effectiveness of policing programs aimed at promoting community connectedness and their impact on reducing violent extremism. To date, there has been no systematic synthesis of the evaluation evidence for these policing approaches and their impact on violent extremism. This is the protocol for a review that will include any policing intervention that aims to promote community connectedness. The present proposed review is necessary to ascertain whether policing interventions that seek to promote community connectedness are effective for reducing violent extremism behaviour, attitudes and beliefs.

## 1 BACKGROUND

### 1.1 The problem, condition or issue

Community engagement and connectedness are identified as potential mitigating factors for those at risk of engaging in violent extremism (Cherney & Hartley, 2017). A focus on inclusion, social connectedness and positive cultural norms is essential for prevention efforts designed to build inclusive communities and weaken the influence of extremist messages and recruiters (Grossman, Peucker, Smith, & Dellal, 2016; Van Den Bos, 2018; Schanzer, Kurzman, Toliver, & Miller, 2016). Growing research suggests that cohesive communities are resilient against violent extremist influences; for example, it is argued that a greater sense of belonging and acceptance can reduce extremist behaviour, attitudes and beliefs (Cherney, Bell, Leslie, Cherney, & Mazerolle, 2018; Grossman et al., 2016; Van Den Bos, 2018).

Police have a critical role in promoting social inclusion and social connectedness and thereby preventing violent extremism. In the general policing literature, police engagement with the public is found to foster a deep understanding of the local communities they police, creating opportunities for improving community relations (Gill, Weisburd, Telep, Vitter, & Bennett, 2014). Strong police‐community relations are likely an important foundation for police being in a position to identify individuals who might be at risk of radicalisation and violent extremism and then work with community leaders to counter the influence of a variety of different types of violent extremist groups including those from the far right, the far left, environmental extremism, political and religious extremism groups. Police are, therefore, key agents in promoting community connectedness, working with community members to build trust, minimise social distancing—particularly amongst culturally diverse communities—and strengthen a sense of belonging by showing that they have the interests of the community at heart (Cherney & Hartley, 2017; Murray, Mueller‐Johnson, & Sherman, 2015).

Increasingly, police are working with a range of different agencies, together actively engaging the community to reduce social isolation, improve economic opportunity and aim to create social and cultural norms that prevent violent extremism (Schanzer et al., 2018). Yet it is unclear whether or not the range of police initiatives that foster community connectedness are able to reduce violent extremism. Thus, it is essential to understand the effectiveness of policing programs aimed at promoting community connectedness and their impact on reducing violent extremism.

### 1.2 The intervention

This review will include any policing intervention that aims to promote community connectedness, which is defined by the presence of two components. First, the intervention must have a *policing* focus, defined as some kind of a strategy, technique, approach, activity, campaign, training, program, directive or funding/organisational change that involves police in some way (other agencies or organisations can be involved; Higginson, Eggins, Mazerolle, & Stanko, 2015). Police involvement is broadly defined as the following.

- Police initiation, development or leadership.
- Police are recipients of the intervention or the intervention is related, focused or targeted to police practices.
- Delivery or implementation of the intervention by police.

Second, the policing intervention must *aim to promote community connectedness*. For the purposes of this review, we define the promotion of community connectedness to mean an intention to increase prosocial linkages or prosocial ties between either community members themselves, community members and police, or community members and people in businesses, houses of worship, schools or any other community‐based organisation. Other terminology that may be used to represent connectedness in the literature includes the following (Thomas, 2019).

- Promotion of common values, norms and/or reciprocity.
- Promotion of social networks, collective efficacy, social cohesion or social capital.
- Promotion of shared problem‐solving or citizen engagement.

We anticipate that policing interventions aiming to promote community connectedness will likely overlap with initiatives labelled community policing (see Gill et al., 2014) or other policing approaches that often aim to enhance community connectedness (e.g., neighbourhood policing or legitimacy approaches). However, we specifically define a policing intervention that aims to promote community connectedness as being characterised by community consultation, partnership or collaboration with citizens and/or organisational entities. Specific strategies may include:

- community meetings or forums;
- developing partnerships with specific organisations (Fox, 2012);
- police liaison programs involving community members (Cherney et al., 2018);
- police work with community leaders to enhance personal skills (e.g., self‐identity, self‐awareness and resilience), employment skills (e.g., teamwork and self‐awareness) or leadership skills (Thomas, 2019);
- routine police work (such as beat policing, foot patrols and community intelligence initiatives) that explicitly seek to promote community connectedness;
- specific initiatives—such as neighbourhood policing teams—that seek to promote community connectedness; or
- police legitimacy enhancing programs that seek to promote a sense of belonging and inclusion within local communities.

### 1.3 How the intervention might work

Police programs that seek to reduce violent extremist behaviours and beliefs through improving community connectedness aim to generate an impact by promoting an increased sense of prosocial belonging and inclusion amongst at‐risk groups. This causal pathway is underpinned by key perspectives in the literature that argue how people are treated by institutional authorities, such as police, has an impact on their sense of identity and belonging by making them feel accepted by broader society (Mazerolle et al., 2014).

Social identity theory and the group value model (see Tyler & Lind 1992) demonstrates that the ways that police engage with citizens will differentially affect the way that people perceive the police and thereby their willingness to comply with directives (see also Bradford, Murphy, & Jackson, 2014; Huo, Smith, Tyler, & Lind, 1996). Some research shows that procedural fairness is more important for those on the margins (De Cremer & Sedikides 2005; Murphy 2013). Other research finds that the procedural justice, social identity and legitimacy pathway is found amongst both those with high and low group identifications (Bradford et al. 2014). Outcomes of police initiatives that build a sense of belonging and inclusion are, therefore, assumed to act as protective factors against radicalisation by ensuring individuals are not influenced by the messaging and grievance narratives that violent extremists use to attract support. We acknowledge, however, Nagin and Telep's (2017) review of the evidence challenging the causal relationship between perceptions of procedurally just treatment of citizens by agents of the criminal justice system and perceptions of police legitimacy. These authors conclude that perceptions of procedurally just treatment are associated with perceptions of police legitimacy and legal compliance (see also Donner, Maskaly, Fridell, & Jennings, 2015; Jackson, Hough, Bradford, & Kuha, 2015; Tyler 2004, 2017; Tyler, Goff, & MacCoun, 2015), yet that these associations are not necessarily reflective of causal connections.

As it relates specifically to tackling violent extremism, the quality of police engagement of with different types of communities—such as the Muslim community—has been shown to influence the degree to which people are willing to partner with police to tackle terrorism (Cherney & Murphy 2017). Police are seen as representatives of the state and when they work with community groups, this helps builds police legitimacy and has a spill over effect on people's sense of belonging and inclusion.

### 1.4 Why it is important to do the review

Community engagement approaches have become a key component of police counterterrorism efforts (Cherney & Hartley, 2017). These strategies have emphasised community engagement and outreach to identify potential violent extremism threats. This has involved police programs that aim to promote collaborative problem solving between police and community members to tackle radicalisation, such as through identifying youth at risk of radicalising to violent extremism (Cherney, 2018). For example, following 9/11, police units in Australia, the United Kingdom, the United States, and Canada were established to undertake outreach with particular community groups (e.g., Muslim communities), with the aim of tackling violence extremism by enhancing relations and connectedness between police and these communities, and also between community members (Cherney, 2018; Ramirez, Quinlan, Malloy, & Shutt, 2013). However, to date, there has been no systematic synthesis of the evaluation evidence for these policing approaches and their impact on violent extremism.^1^ Therefore, the present proposed review is necessary to ascertain whether policing interventions that seek to promote community connectedness are effective for reducing violent extremism behaviour, attitudes and beliefs. In addition, the results from this review will inform future decision making relating to both the design and evaluation of police programs by identifying potential gaps in the evidence‐base and level of investment needed in evaluation of primary studies.

## 2 OBJECTIVES

The primary objective of this review is to answer the question: how effective are police programs that seek to increase community connectedness for reducing violent extremism attitudes, beliefs and behaviours? If there are sufficient data, the review will also examine whether the effectiveness of these interventions vary by the following factors: geographical location, target population and type of policing strategy used to promote connectedness.

## 3 METHODOLOGY

### 3.1 Criteria for including and excluding studies

#### 3.1.1 Types of study designs

This review will include quantitative impact evaluations that utilise a randomised experimental (e.g., RCTs) or a quasi‐experimental design with a comparison group that does not receive the intervention. We will include studies where the comparison group receives “business‐as‐usual” policing, no intervention or an alternative intervention (treatment–treatment designs).

Although not as robust as RCTs, “strong” quasi‐experiments can be used to provide causal inference when there are elements of the design that aim to minimise threats to internal validity (see Farrington, 2003; Shadish, Cook, & Campbell, 2002). Minimising threats to internal validity can include controlling case assignment to treatment and control groups (regression discontinuity), matching characteristics of the treatment and control groups (matched control), statistically accounting for differences between the treatment and control groups (designs using multiple regression analysis) or providing a difference‐in‐difference analysis (parallel cohorts with pretest and posttest measures). Therefore, we will include the following “strong” quasi‐experimental designs in this review.

- Cross‐over designs.
- Regression discontinuity designs.
- Designs using multivariate controls (e.g., multiple regression).
- Matched control group designs with or without preintervention baseline measures (propensity or statistically matched).
- Unmatched control group designs without preintervention measures where the control group has face validity.
- Unmatched control group designs with pre–postintervention measures which allow for difference‐in‐difference analysis.
- Short interrupted time‐series designs with control group^2^ (less than 25 pre‐ and 25 postintervention observations; Glass, 1997).
- Long interrupted time‐series designs with or without a control group (≥25 pre‐ and postintervention observations; Glass, 1997).

Weaker quasi‐experimental designs can be used to demonstrate the magnitude of the relationship between an intervention and an outcome. However, we will exclude the following weaker quasi‐experimental designs due to their limitations in establishing causality.

- Raw unadjusted correlational designs where the variation in the level of the intervention is compared with the variation in the level of the outcome.
- Single group designs with pre‐ and postintervention measures.

#### 3.1.2 Types of participants

This review will consider the impact of community connectedness policing interventions on the following population subjects.

- Individuals of any age, gender or ethnicity.
- Microplaces (e.g., street corners, buildings, police beats and street segments).
- Macroplaces (e.g., neighbourhoods or larger geographies).

We will place no limits on the geographical region reported in the study. Specifically, we will include studies conducted in high‐, low‐ and middle‐income countries in the review.

#### 3.1.3 Types of interventions

This review will include any policing intervention that aims to promote community connectedness. Specifically, each study must meet the following two intervention criteria.

1. Report on a *policing intervention*, defined as some kind of a strategy, technique, approach, activity, campaign, training, program, directive or funding/organisational change that involves police in some way (other agencies or organisations can be involved; Higginson et al. 2015). Police involvement is broadly defined as:
  - police initiation, development or leadership;
  - Police are recipients of the intervention or the intervention is related, focused or targeted to police practices; or
  - delivery or implementation of the intervention by police.

2. Report on a policing intervention that *aims to promote community connectedness*. For the purposes of this review, we define the promotion of community connectedness to mean an intention to increase linkages or ties between either the community members themselves or community members and police. Other terminology that may be used to represent connectedness in the literature includes (Thomas, 2019):
  - promotion of common values, norms and/or reciprocity;
  - promotion of social networks, collective efficacy, social cohesion and/or social capital; or
  - promotion of shared problem‐solving or citizen engagement.

We anticipate that policing interventions aiming to promote community connectedness will include, more generally, community consultation, partnership, or collaboration with citizens and/or organisational entities. Specific strategies may include (but are not limited to):

- community meetings or forums;
- developing partnerships with specific organisations (Fox, 2012);
- police liaison programs involving community members (Cherney et al., 2018); or
- police work with community leaders to enhance personal skills (e.g., self‐identity, self‐awareness and resilience), employment skills (e.g., teamwork and self‐awareness) or leadership skills (Thomas, 2019).

#### 3.1.4 Types of outcome measures

Terrorism is one outcome of violent extremism, which constitutes both a cognitive and behavioural component. In the literature, a distinction is made between radicalisation as constituting beliefs, while violent extremism is the behavioural outcome of those beliefs. In this review, will include programs focused on individuals and groups identified as at risk of radicalisation due to beliefs and or associations, as well as those who have acted on those beliefs. This is to ensure we capture police programs tackling different levels of violent extremism.

Hence, this review will include studies where the measured outcome is violent extremist attitudes, beliefs and behaviour. For the purposes of this review, violent extremism is defined as “advocating, engaging in, preparing or otherwise supporting ideologically motivated or justified violence to further social, economic and political objectives” (Barker, 2015; Horgan, 2009; Khalil and Zeuthen, 2016; United States Agency for International Development, 2016).

It is important to note that violent extremism is defined and captured differently across countries (e.g., Barker, 2015; Government Offices of Sweden, 2011; Lowe, 2017; Norwegian Ministry of Justice and Public Security, 2014; Public Safety Canada, 2018). In order to capture research on violent extremism across global contexts, we will use either the study authors' definitions of outcomes or, in the case of ambiguity, consult definitions used in each country's associated terrorism legislation or policies to guide the inclusion of studies with potentially eligible outcomes.

The review will also include studies where the outcome is disengagement and/or deradicalisation, which are often encompassed within conceptualisations of violent extremism (Klausen, Campion, Needle, Nguyen, & Libretti, 2016). Disengagement generally captures the behavioural aspect of extremism and refers to reducing or ceasing physical involvement in violent or radical activities (Horgan, 2009). In contrast, deradicalisation is defined as the psychological shift in attitudes or beliefs (Horgan & Braddock, 2010). This can encompass a variety of ideologies, including: Islamist (or jihadist), far‐right (right‐wing), far‐left (left‐wing) and single issue (anti‐abortion, animal liberationists; National Consortium of the Study of Terrorism and Responses to Terrorism [START], 2018).

We will include outcome data that are measured through self‐report instruments, interviews, observations and/or official data (e.g., arrests or convictions). Some examples of how violent extremism attitudes, beliefs and behaviour can be measured include:

- official data taken from the Profiles of Radicalised Individuals in the United States (PIRUS),^3^ which includes: active participation in operational plots intending to cause causalities (e.g., gathering weapons, choosing targets), recruiting individuals to an official or unofficial extremist group and providing material/financial support to extremist organisations (START, 2018);
- level of disillusionment with, disappointment with or renouncement of extremist group members, extremist leaders and/or radical ideology (Barrelle, 2015; Berger, 2016; San, 2018);
- willingness to engage in violence (San, 2018);
- modification of group and personal social identity (Barrelle, 2015);
- level of acceptance and/or engagement with cultural and religious differences or pluralistic views (Barrelle, 2015);
- number and/or strength of ties with extremist social networks, extremist recruiters (Perliger & Pedahzur, 2011); and/or
- amount of extremist activity (San, 2018).

#### 3.1.5 Duration of follow‐up

Studies will be included regardless of the length of follow‐up after the intervention. If the length of follow‐up varies across studies, we will group and synthesise studies with similar follow‐up durations. For example, short (e.g., 0–3 months after intervention), medium (>3 months, <6 months), and long‐term follow‐up (>6 months after intervention).

#### 3.1.6 Types of settings

We will include studies reporting on an impact evaluation of an eligible intervention using eligible participants, outcome(s) and an eligible research design in any setting. Where there are multiple conceptually distinct settings, we will synthesise the studies within the settings separately.

We will include studies written in any language that are identified in the search. We will use Google Translate for the title and abstract screening stage to identify whether a non‐English language study is potentially eligible for review, and will call upon our international network of colleagues for assistance with full‐text screening and coding where needed.

We will include studies published between 2002 and 2018 in the review.

### 3.2 Search strategy

The search for this review will be led by the Global Policing Database (GPD) research team at the University of Queensland (Elizabeth Eggins and Lorraine Mazerolle) and Queensland University of Technology (Angela Higginson). The University of Queensland is home to the GPD (see http://www.gpd.uq.edu.au), which will serve as the main search location for this review. The GPD is a web‐based and searchable database designed to capture all published and unpublished experimental and quasi‐experimental evaluations of policing interventions conducted since 1950. There are no restrictions on the type of policing technique, type of outcome measure or language of the research (Higginson et al., 2015). The GPD is compiled using systematic search and screening techniques, which are reported in Higginson et al. (2015) and summarised in Appendices A and B. Broadly, the GPD search protocol includes an extensive range of search locations to ensure that both published and unpublished research is captured across criminology and allied disciplines.

Because the GPD includes experimental and quasi‐experimental studies that evaluate interventions relating to police or policing, with no limits on outcome measures, we will use a broad search to capture studies for the review. Specifically, we will search the title and abstracts of the corpus of GPD full‐text documents that have been classified as reporting on a quantitative impact evaluation of a policing intervention between 2002 and 2018 using the following search terms: *terror* OR extrem* OR *radical* (Table 1).

**Table 1:** Grey literature search locations

Organisation	Website
Global Terrorism Research Centre (Monash University)	http://artsonline.monash.edu.au/gtrec/publications/
Triangle Centre on Terrorism and Homeland Security	https://sites.duke.edu/tcths/#
Department of Homeland Security	https://www.dhs.gov/topics
Public Safety Canada	https://www.publicsafety.gc.ca/index‐en.aspx
National Consortium for the Study of Terrorism and Responses to Terrorism (START)	https://www.start.umd.edu/
Terrorism Research Centre	http://www.terrorism.org/
Global Centre on Cooperative Security	https://www.globalcenter.org/publications/
Hedayah	http://www.hedayahcenter.org/publications
RAND Corporation	https://www.rand.org/topics/terrorism.html?content‐type=research
Radicalisation Awareness Network (RAN)	https://ec.europa.eu/home‐affairs/what‐we‐do/networks/radicalisation_awareness_network_en
RadicalisationResearch	https://www.radicalisationresearch.org/
Royal United Services Institute (RUSI)	https://rusi.org/
Impact Europe	http://impacteurope.eu/

We will also employ additional strategies to extend the GPD search. This includes the following.

- Searching trial registries (as listed by WHO https://www.who.int/ictrp/network/primary/en/) and Office for Human Research Protections (https://www.hhs.gov/ohrp/international/clinical‐trial‐registries/index.html).
- Searching counterterrorism organisation websites (see Table 1).
- Conducting reference harvesting on both the corpus of eligible documents and existing reviews.
- Forward citation searching for all eligible documents.
- Liaising with the Five Country Research and Development Network (5RD), and the Department of Homeland Security Advisory Board network for the Campbell Collaboration grants, to enquire about eligible studies that may not be publicly available.
- Personally contacting prominent scholars in the field and authors of eligible studies to enquire about eligible studies not yet disseminated or published.
- Hand‐searching the four most recent issues of the following journals to identify eligible documents yet to be indexed in academic databases:
  a. Critical Studies on Terrorism;
  b. Dynamics of Asymmetric Conflict;
  c. Intelligence and Counter Terrorism;
  d. International Journal of Conflict and Violence;
  e. Journal for Deradicalization;
  f. Journal of Policing;
  g. Perspectives on Terrorism;
  h. Police Quarterly;
  i. Policing—An international Journal of Police Strategies and Management;
  j. Policing and Society;
  k. Sciences of Terrorism and Political Aggression;
  l. Studies in Conflict & Terrorism;
  m. Terrorism and Political Violence.

### 3.3 Description of methods used in primary research

Existing literature highlights the burgeoning range of policing approaches that focuses on increasing community connectedness in the context of terrorism, radicalisation and/or single issue violent extremism (e.g., see Cherney & Murphy, 2017; Downing, 2009; Schanzer et al., 2016; Silk, 2012). Based on this literature, we anticipate that the vast majority of studies captured by our review will utilise quasi‐experimental research designs with some conceptual differences in the measurement of outcome variables.

### 3.4 Criteria for determination of independent findings

Issues of dependence can arise where (a) multiple documents report on a single empirical study, (b) multiple conceptually‐similar outcomes are reported in the one document and/or (c) studies have clustering in their research design. For meta‐analyses, each eligible study will be included only once for each conceptually distinct outcome category.

The software that will be used for this review allows the nesting of multiple dependent documents relating to a single study, to allow for the identification of multiple reports of the same study. In cases where there are dependent documents reporting on the one study, all documents will be coded and the most complete report of the study will be used for data extraction. If necessary and where appropriate, data may be extracted from multiple documents to enable the calculation of effect sizes.

If documents report on multiple conceptually similar outcomes, these effect sizes will be averaged and only the averaged effect size will be included in the meta‐analysis (Borenstein, Cooper, Hedges, & Valentine, 2009). Where studies utilise a research design with clustering (e.g., study sites assigned to conditions), the method proposed by Fu et al. (2013) and Higgins et al. (2011) will be used to adjust the standard error (SE). Where documents do not report the required intraclass correlation coefficient (ICC), sensitivity analyses will be conducted to explore whether the results of the meta‐analyses change with ICCs of 0, 0.03, 0.02 and 0.1 (Barlow, Bergman, Kornør, Wei, & Bennett, 2016).

### 3.5 Details of study coding categories

#### 3.5.1 Title and abstract screening

The first stage of assessing study eligibility will begin with title and abstract screening of all unique records identified in the systematic search. After removing duplicates and ineligible document types (e.g., book reviews, blog posts) from the results of the systematic search, all records will be imported into the review management software, *SysReview* (Higginson & Neville, 2014). Each title and abstract (record) will then be assessed according to the following exclusion criteria.

1. Ineligible document type.
2. Document is not unique.
3. Document is not about terrorism or extremism.

Although all efforts will be made to remove ineligible document types and duplicates prior to screening, automated and manual cleaning can be less than perfect. As such, the first two exclusion criteria will be used to remove ineligible document types and duplicates prior to screening each record on substantive content relevance.

Most records indexed in the GPD have a pre‐existing full‐text document. However, records from the additional searches that are deemed as potentially eligible at the title and abstract screening stage will progress to literature retrieval, where the full‐text document will be located. Where full‐text documents cannot be retrieved via existing university resources, they will be ordered through the university libraries of the review authors or by contacting study authors. All potentially eligible records will then progress to full‐text eligibility screening.

#### 3.5.2 Full‐text eligibility screening

The full‐text of each document will be screened for final eligibility according to the following exclusion criteria.

1. Ineligible document type.
2. Document is not unique.
3. Document does not evaluate a policing intervention that aims to promote community connectedness.
4. The evaluation does not report violent extremism attitudes, beliefs or behaviour as an outcome.

All efforts will be made to remove ineligible document types and duplicate documents in earlier stages. However, sometimes these types of records can progress into later stages of screening (e.g., where duplicate records are not adjacent during screening or where screeners cannot unambiguously determine whether a record is ineligible based on the title and abstract). Therefore, the first two exclusion criteria will be used to remove ineligible document types and duplicates.

#### 3.5.3 Full‐text coding and risk of bias assessment

Eligible documents progressing from the full‐text screening stage will be coded within *SysReview*, using the coding companion provided in Appendix C. Broadly, studies will be coded according to the following domains.

1. General study characteristics (e.g., document type, study location).
2. Participants (e.g., sample characteristics by condition).
3. Intervention (e.g., intervention components, intensity and setting).
4. Outcomes (e.g., conceptualisation, mode of measurement and time‐points).
5. Research methodology (e.g., design, unit and type of assignment).
6. Effect size data.
7. Risk of bias.

Risk of bias will be evaluated using either the Cochrane randomised or nonrandomised risk of bias tools. Using these tools, studies will be rated across domains as having high, low or unclear risk of bias. Study authors will be approached to obtain missing data where a domain is rated as “unclear”. Results of the risk of bias assessment will be presented in summary tables and in a risk of bias summary figure. Depending on available data, sensitivity analysis will be used to examine the impact of risk of bias on effect estimates and corresponding confidence intervals. Possible analyses will include forest plots stratified by level of risk, moderator analysis or meta‐regression. The level of variation in risk of bias across included studies will determine the approach to incorporate risk of bias into statistical analyses. For example, statistical analysis may be stratified by level of risk or all studies may be included in one analysis with a narrative discussion of the risk of bias (see Higgins et al., 2011 for more details).

### 3.6 Statistical procedures and conventions

Meta‐analyses will be performed for all outcomes where there are at least two independent effect sizes, and we will conduct separate meta‐analyses for each set of conceptually similar outcomes. We will also conduct separate meta‐analyses for studies where participants are individuals and studies where participants are places, even if both groups of studies report on the same outcome. Random effects inverse variance meta‐analyses (Lipsey & Wilson, 2001) will be conducted in R using *rmeta* (Lumley, 2015; available at https://CRAN.R‐project.org/package=rmeta). Mean effect sizes and their corresponding confidence intervals will be reported in‐text and in graphical forest plots.

Where the participants are individuals, we anticipate that evaluations will typically report outcomes as continuous measures (e.g., willingness to engage in violence, self‐reported on a Likert scale), and in these instances, Hedges' *g* (standardised mean differences [SMDs]) will be computed. Where evaluations report binary outcomes (e.g., disengagement from radicalised peers: yes/no), effect sizes will be computed as odds ratios and then transformed into Hedges' *g* for meta‐analyses (see Borenstein, Hedges, Higgins, & Rothstein, 2009). Throughout the review, we will follow Campbell Collaboration guidelines and aim to transform the smallest number of effect sizes to a common effect size (Polanin & Snilstveit, 2016), therefore the final effect size metric will be that which is calculated most commonly for each outcome.

Where the participants are micro‐ or macroplaces, we anticipate that evaluations may report outcomes as counts or rates in the intervention and comparison areas, before and after the intervention (e.g., number of radicalised individuals). In these instances, we will calculate the effect size as the relative incident rate ratio (RIRR), which can be interpreted as the relative proportion change in the outcome in the treatment area after the intervention, compared with the comparison area (Farrington, Gill, Waples, & Argomaniz, 2007; Higginson & Mazerolle, 2014). RIRR is calculated as

RIRR=ad/bc,

where *a* denotes the count or rate in the intervention area before the intervention, *b* denotes the count or rate in the intervention area after the intervention, *c* denotes the count or rate in comparison area before the intervention, and *d* denotes the count or rate in the comparison area after the intervention. The RIRR will be converted to a log relative incident rate ratio (LRIRR) for synthesis, but converted back to RIRR for more intuitive reporting. The standard error of the LRIRR is initially calculated as

$$\mathrm{se}\left(\text{LRIRR}\right)=\sqrt{\frac{1}{a}+\frac{1}{b}+\frac{1}{c}+\frac{1}{d}},$$

however, the odds ratio formula of the RIRR assumes a Poisson distribution, to which crime data typically does not conform. Therefore, following Wilson, Olaghere, & Kimbrell (2019) we will calculate an overdispersion parameter (*ϕ*) which is a linear function of the mean, assuming a quasi‐Poisson distribution:

$$\emptyset =\frac{s^{2}}{\bar{y}},$$

where *s* is the standard deviation and $\overset{®}{y}$ is the mean of the observations. If *∅* > 1, we will adjust the standard error by multiplying the standard error by the quasi‐Poisson overdispersion parameter *∅*.

We will conduct separate meta‐analyses for each of the reported follow‐up time points, where data permit. Where studies report multiple points of follow‐up, effect sizes will be calculated for each time‐point, but synthesised separately with studies that have similar outcome time‐points. If studies report both baseline and postintervention outcome data, SMDs will be calculated using baseline adjusted mean differences (i.e., mean change scores) and the change score standard deviations will be standardised using the raw standard deviation within groups. If the standard deviation for mean change scores is not available, we will follow Lipsey and Wilson's (2001) formula to calculate the standard deviation. If studies report follow‐up outcome data, post‐only outcome data will be used to estimate SMDs, and follow‐up outcomes will be analysed separately from postintervention outcomes.

For each meta‐analysis, we will assess heterogeneity in effect sizes using the *I* ^2^ statistic, *χ* ^2^ test, and *τ* ^2^ (Higgins & Thompson, 2002). We will conduct moderator analyses to explore potential sources of heterogeneity, using the variables listed under “Objectives” section. The analogue to analysis of variance (ANOVA) will be used for categorical moderators and regression‐based approaches will be used for continuous moderators. We may also conduct additional exploratory subgroup analyses, but will clearly distinguish between a priori and post hoc analyses in our reporting.

Finally, we will visually and statistically assess the data for evidence of publication bias. We will inspect funnel plots for asymmetry, and if asymmetry is detected, we will conduct subgroup analyses to assess if the effect sizes from the published and unpublished documents are significantly different.

### 3.7 Treatment of qualitative research

This review will not include qualitative research.

## ROLES AND RESPONSIBILITIES

- Content: Mazerolle, Cherney, Belton, Eggins, Higginson, Hine.
- Systematic review methods: Eggins, Higginson, Hine.
- Statistical analysis: Higginson, Eggins.
- Information retrieval: Eggins, Higginson, Hine.

Lorraine Mazerolle is a Professor of Criminology with the School of Social Science at the University of Queensland and currently the Co‐Chair of the Campbell Collaboration Crime and Justice Coordinating Group. She has won numerous US and Australian competitive research grants—including systematic reviews—on topics such as third‐party policing, police engagement with high risk people and disadvantaged communities, community regulation, problem‐oriented policing, police technologies, civil remedies, street‐level drug enforcement and policing public housing sites.

Adrian Cherney is an Associate Professor with the School of Social Science at the University of Queensland and an Australian Research Council (ARC) Future Fellow. His current research focuses on the evaluation of programs aimed at countering violent extremism and he has undertaken research on the supervision of terrorist offenders in Australian who have been released into the community on parole. His ARC Future Fellowship aims to develop and test metrics and methods to evaluate case‐managed interventions targeting individuals who have been charged for a terrorist offence or have been identified as at risk of radicalising to violent extremism. He has secured both international and national competitive grants.

Ms Elizabeth Eggins is the Managing Editor of the Campbell Collaboration Crime and Justice Coordinating Group. She has coauthored and managed multiple systematic reviews in criminology and social welfare disciplines, with particular expertise in methodology, analysis and information retrieval.

Dr Angela Higginson is a Senior Lecturer with the School of Justice, Faculty of Law, QUT and current editor of the Campbell Collaboration Crime and Justice Coordinating Group. She is an ARC Discovery Early Career Research Award (DECRA) fellow for 2018‐2020, and her DECRA project examines the correlates and consequences of ethnically motivated youth hate crime in Australia. Much of Angela's work has focused on policing and community processes for crime control, with a particular expertise in evaluation through systematic reviews and meta‐analysis.

Ms Lorelei Hine is a Research Assistant with the School of Social Science at the University of Queensland. She has assisted with the project management of several systematic reviews as well as the Global Policing Database. This has provided her with expertise in both systematic review methodology and substantive content in relation to criminal justice interventions.

Ms Emma Belton is a PhD student and Research Assistant with the School of Social Science at the University of Queensland. She has coauthored and worked on projects in the area of countering violent extremism (CVE), including the collection and analysis of data and evaluations of programs aimed CVE.

## CONFLICT OF INTERESTS

Three of the review authors have internal roles within the Campbell Collaboration Crime and Justice Group. Lorraine Mazerolle is the Co‐Chair of the Crime and Justice Coordinating Group (CJCG), Angela Higginson is the Editor of the CJCG and Elizabeth Eggins is the Managing Editor of the CJCG. Consequently, Mazerolle, Higginson and Eggins will not be involved in any editorial or internal Campbell Collaboration communications about this review. In addition, Adrian Cherney has published research that is closely linked with the review topic. To minimise potential bias, Cherney will not be involved in the screening or coding of any studies for this review.

## PRELIMINARY TIMEFRAME

The final review is due for submission on December 20, 2019.

## PLANS FOR UPDATING THE REVIEW

Lorraine Mazerolle and Adrian Cherney will be responsible for updates of this review, which are anticipated to occur every 3 to 5 years.

## Figures and Tables

**Figure B1:**
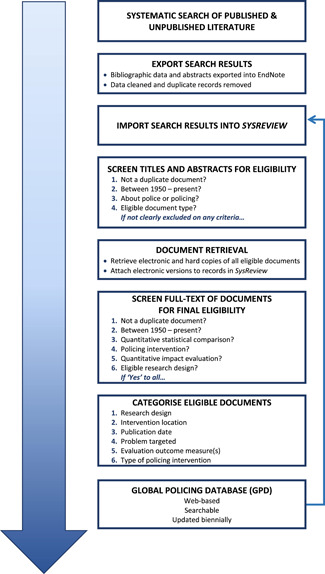
GPD systematic compilation process. GPD, Global Policing Database

## Footnotes

^1^
We conducted a search of the literature using the following terms to identify existing reviews: terroris* OR extremis* OR radicali*. Searches of the Campbell Collaboration library, Cochrane Collaboration library, PROSPERO register and Google Scholar did not identify any existing systematic reviews (completed or ongoing) on the specific topic proposed in this protocol.

^2^
We will include all short interrupted time‐series designs with control group, as long as the design includes a minimum of one preintervention observation for each of the treatment and comparison groups. This approach is consistent with the inclusion of unmatched control group designs with pre–post intervention measures. For studies with extremely short preintervention time series (less than four pre‐ and four postintervention observations), the data will be collapsed and treated as pre–post averages, rather than as true time series data.

^3^
We use the term “official data” loosely here as there is limited official data or direct measures of violent extremism. PIRUS stipulates strict inclusion criteria of individuals who have been radicalised or radicalised to violence.

## APPENDIX A GPD SYSTEMATIC SEARCH STRATEGY

### Search terms

To ensure optimum sensitivity and specificity, the GPD search strategy utilises a combination of free‐text and controlled vocabulary search terms. Because controlled vocabularies and search capabilities vary across databases, the exact combination of search terms and field codes are adapted to each database. Final search syntax for each location will be reported in the final review.

The free‐text search terms for the GPD are provided in Table A1 and are grouped by substantive (i.e., some form of policing) and evaluation terminology. Although the search strategy may vary slightly across search locations, it follows a number of general rules, which are as follows.

- Search terms are combined into search strings using Boolean operators “AND” and “OR”. Specifically, terms within each category are combined with “OR”, and categories will be combined with “AND”. For example: (police OR policing OR “law#enforcement”) AND (analy* OR ANCOVA OR ANOVA OR …).
- Compound terms (e.g., law enforcement) are considered single terms in search strings by using quotation marks (i.e., “law*enforcement”) to ensure that the database searches for the entire term rather than separate words.
- Wild cards and truncation codes are used for search terms with multiple iterations from a stem word (e.g., evaluation, evaluate) or spelling variations (e.g., evaluat* or randomi#e).
- If a database has a controlled vocabulary term that is equivalent to “POLICE”, the term is combined in a search string that includes both the policing and evaluation free‐text search terms. This approach ensures that the search retrieves documents that do not use policing terms in the title/abstract but have been indexed as being related to policing in the database. An example of this approach is the following search string: (((SU: “POLICE”) OR (TI,AB,KW: police OR policing OR “law*enforcement”)) AND (TI,AB,KW: intervention* OR evaluat* OR compar* OR …)).
- For search locations with limited search functionality, a broad search that uses only the policing free‐text terms is implemented.
- Multidisciplinary database searches are limited to relevant disciplines (e.g., include social sciences but exclude physical sciences).
- Search results are refined to exclude specific types of documents that are not suitable for systematic reviews (e.g., newspapers, front/back matter, book reviews).

**Table A1:** Free‐text search terms for the GPD systematic search

Policing search terms	Evaluation search terms			
police	analy*	data	outcome*	result*
policing	ANCOVA	effect*	paramet*	“risk#ratio*”
“law*enforcement”	ANOVA	efficacy	“post‐test”	sampl*
constab*	“ABAB design”	eval*	posttest	“standard deviation*”
detective*	“AB design”	experiment*	“post test”	statistic*
sheriff*	baseline	hypothes*	predict*	studies
	causa*	impact*	“pre‐test”	study
	“chi#square”	intervent*	pretest	survey*
	coefficient*	interview*	program*	“systematic review*”
	“comparison condition*”	longitudinal	“propensity score*”	“t#test*”
	“comparison group*”	MANCOVA	quantitative	“time#series”
	“control condition*”	MANOVA	“quasi#experiment*”	treatment*
	“control group*”	“matched group”	questionnaire*	variable*
	correlat*	measure*	random*	variance
	covariat*	“meta‐analy*”	RCT	
	“cross#section*”	“odds#ratio*	regress*	

### Search locations

To reduce publication and discipline bias, the GPD search strategy adopts an international scope and involves searching for literature across a number of disciplines (e.g., criminology, law, political science, public health, sociology, social science and social work). The search captures a comprehensive range of published (i.e., journal articles, book chapters and books) and unpublished literature (e.g., working papers, governmental reports, technical reports, conference proceedings and dissertations) by implementing a search strategy across bibliographic/academic, grey literature and dissertation databases or repositories.

It is noted that there is substantial overlap of the content coverage between many of the databases. Therefore, the *Optimal Searching of Indexing Databases* (OSID) computer program (Higginson & Neville, 2014) has been used to analyse the content crossover for all databases that have accessible content coverage lists. OSID analyses the content coverage and creates a search location solution that provides the most comprehensive coverage via the least number of databases. Another advantage of using OSID when designing a search strategy is the reduction in the number of duplicates that would need to be removed prior to the screening phase. Databases with more than 10 unique titles are searched in full, whereas databases with ≤10 unique titles were searched only the unique titles and any nonserial content (e.g., reports, conference proceedings). Where a modified search of a database would be more labour‐intensive than a full search and export results, a full search of the database is conducted. The final search locations and solutions are reported in Table A2.

**Table A2:** GPD search locations and protocol (January 1, 1950–December 2018)

Indexed and academic databases		Content coverage fed into OSID?	Full or modified search?	Search modifications
ProQuest	Criminal Justice	Yes	Full	None
	Dissertation and Theses Database Global	Not available	Modified	Social Sciences subset
	Political Science	Yes	Full	None
	Periodical Archive Online	Yes	Full	None
	Research Library	Yes	Modified	Social Sciences subset
	Social Science Journals	Yes	Full	None
	Sociology	Yes	Modified	Search 2 unique journal titles and non‐serial content only
	Applied Social Sciences Index and Abstracts	Yes	Full	None
	International Bibliography of the Social Sciences	Yes	Full	None
	Public Affairs Information Service	Yes	Full	None
	Social Services Abstracts	Yes	Modified	Search 5 unique journal titles and non‐serial content only
	Sociological Abstracts	Yes	Full	None
	Worldwide Political Sciences Abstracts	Yes	Modified	Search 9 unique journal titles and non‐serial content only
EBSCO	Academic Search Premier	Yes	Full	None
	Criminal Justice Abstracts	Yes	Full	None
	EconLit	Yes	Full	None
	MEDLINE with Full‐Text	Yes	Full	None
	Social Sciences Full‐Text	Yes	Full	None
OVID	International Political Science Abstracts	Not available	Full	None
	PsycARTICLES	Yes	Modified	Search four unique journal titles only
	PsycEXTRA	Not Available	Full	None
	PsycINFO	Yes	Full	None
	Social Work Abstracts	Not Available	Full	None
Web of Science	Current Contents Connect—Social and Behavioural Sciences Edition	Yes	Modified	Search one unique journal title and nonserial content only
	Book Citation Index (Social Sciences and Humanities)	Not Available	Full	None
	Conference Proceedings Citation Index (Social Sciences and Humanities)	Not available	Full	None
	Social Science Citation Index	Yes	Full	None
Informit	Australian Attorney General Information Service	Yes	Full	None
	Australian Criminology Database (CINCH)	Yes	Full	None
	Australian Federal Police Database	Yes	Full	None
	Australian Public Affairs Full‐Text	Yes	Full	None
	DRUG	Yes	Full	None
	Health & Society Database	Yes	Modified	Search unique journal titles and nonserial content only
	Humanities and Social Sciences Collection	Yes	Full	None
Gale‐Cengage	Expanded Academic ASAP	Yes	Full	None
Standalone and open access databases	Cambridge Journals Online	Yes	Modified	Search 4 unique journal titles in Law and Political Science collections and full search of Social Studies collection
	Directory of Open Access Journals	Yes	Full	None
	HeinOnline	Yes	Modified	Law Journals Online collection only
	JSTOR	Yes	Modified	Search unique titles across the Law, Political Science, Public Health, Public Policy, Social Work and Sociology collections only. The Criminal Justice collection had no unique content and so will be excluded from the search. Only 10% of content in this database have abstracts and a full‐text search returns >250,000 results because of inability to construct complex search strings. Therefore, a modified search of the unique titles across these collections will be more pragmatic than a full search of the database
	Oxford Scholarship Online	Yes	Full	None
	Sage Journals Online and Archive (Sage Premier)	Yes	Modified	Search 5 unique journal titles and non‐serial content only.
	ScienceDirect	Yes	Full	None
	SCOPUS	Yes	Full	None
	SpringerLink	Yes	Full	Although this database has low uniqueness when combined with the full set of databases, a full search using only the policing search terms will be more pragmatic than a modified search on unique titles because of the restricted search functionality of this database
	Taylor & Francis Online	Yes	Modified	Although this database has low uniqueness when combined with the full set of databases, a full search using only the policing search terms will be more pragmatic than a modified search on unique titles because of the restricted search functionality of this database.
	Wiley Online Library	Yes	Full	None
	California Commission on Peace Officer Standards & Training Library	No	Full	None
	Cochrane Library	No	Full	None
	CrimeSolutions.gov	No	Full	None
	Database of Abstracts of Reviews of Effectiveness (DARE)	No	Full	None
	FBI – The Fault (Reports and Publications)	No	Full	None
	Evidence‐Based Policing Matrix	No	Full	None
	International Initiative for Impact Evaluation Database (3ie)	No	Full	None
	National Criminal Justice Reference Service	No	Full	None
	Safety Lit Database	No	Full	None
	Australian Institute of Criminology	No	Full	None
	Bureau of Police Research and Development (India)	No	Full	None
	Canadian Police Research Catalogue	No	Full	None
	Centre for Problem‐Oriented Policing	No	Full	None
	College of Policing (including POLKA and Crime Reduction Toolkit)	No	Full	None
	European Police College (CEPOL)	No	Full	None
	Evidence for Policy and Practice Information and Coordinating Centre	No	Full	None
	National Research Institute of Police Science (Japanese)	No	Full	None
	Office of Community Oriented Policing Services	No	Full	None
	Police Executive Research Forum (US)	No	Full	None
	Police Foundation (US)	No	Full	None
	Tasmania Institute of Law Enforcement Studies (Australia)	No	Full	None
	Policing Online Information System (POLIS, Europe)	No	Full	None
	Scottish Institute for Policing Research	No	Full	None
	Centre of Excellence in Policing and Security (Australian, now archived)	No	Full	None

## APPENDIX B GPD SYSTEMATIC COMPILATION STRATEGY

### Inclusion criteria

Each record captured by the GPD systematic search must satisfy all inclusion criteria to be included in the GPD: timeframe, intervention and research design. There are no restrictions applied to the types of outcomes, participants, settings or languages considered eligible for inclusion in the GPD.

#### Types of interventions

Each document must contain an impact evaluation of a policing intervention. Policing interventions are defined as some kind of a strategy, program, technique, approach, activity, campaign, training, directive or funding/organisational change that involves police in some way (other agencies or organisations can be involved). Police involvement is broadly defined as following.

- Police initiation, development or leadership.
- Police are recipients of the intervention or the intervention is related, focused or targeted to police practices.
- Delivery or implementation of the intervention by police.

#### Types of study designs

The GPD includes quantitative impact evaluations of policing interventions that utilise randomised experimental (e.g., RCTs) or quasi‐experimental evaluation designs with a valid comparison group that does not receive the intervention. The GPD includes designs where the comparison group receives “business‐as‐usual” policing, no intervention or an alternative intervention (treatment‐treatment designs).

The specific list of research designs included in the GPD are as follows.

- Systematic reviews with or without meta‐analyses.
- Cross‐over designs.
- Cost‐benefit analyses.
- Regression discontinuity designs.
- Designs using multivariate controls (e.g., multiple regression).
- Matched control group designs with or without preintervention baseline measures (propensity or statistically matched).
- Unmatched control group designs with pre–post intervention measures which allow for difference‐in‐difference analysis.
- Unmatched control group designs without preintervention measures where the control group has face validity.
- Short interrupted time‐series designs with control group (<25 pre‐ and 25 postintervention observations).
- Long interrupted time‐series designs with or without a control group (≥25 pre‐ and postintervention observations).
- Raw unadjusted correlational designs where the variation in the level of the intervention is compared to the variation in the level of the outcome.

The GPD excludes single‐group designs with pre‐ and postintervention measures as these designs are highly subject to bias and threats to internal validity.

### Systematic screening

To establish eligibility, records captured by the GPD search are progress through a series of systematic stages which are summarised in Figure B1, with additional detail provided in the following subsections.

All research staff working on the GPD undergo standardised training before beginning work within any of the stages detailed below. Staff then complete short training simulations to enable an assessment of their understanding of the GPD protocols and highlight any areas for additional training. In addition, random samples of each staff's work are regularly cross checked to ensure adherence to protocols. Disagreements about screening decisions between staff are mediated by either the project manager or GPD chief investigators.

#### Title and abstract screening

After removing duplicates, the title and abstract of records captured by the GPD systematic search is screened by trained research staff to identify potentially eligible research that satisfies the following criteria.

- Document is dated between 1950 and present.
- Document is unique (i.e., not a duplicate).
- Document is about police or policing.
- Document is an eligible document type (e.g., not a book review).

Records are excluded if the answer to any one of the criteria is unambiguously “No”, and will be classified as potentially eligible otherwise. Records classified as eligible at the title and abstract screening stage progress to full‐text document retrieval and screening stages.

#### Full‐text eligibility screening

Wherever possible, a full‐text electronic version of an eligible record is imported into *SysReview* (review management software; Higginson & Neville, 2014). For records without an electronic version, a hardcopy of the record is located to enable full‐text eligibility screening. The full‐text of each document is screened to identify studies that satisfy the following criteria.

- Document is dated between 1950 and present.
- Document is unique.
- Document reports a quantitative statistical comparison.
- Document reports on policing evaluation.
- Document reports in a quantitative impact evaluation of a policing intervention.
- Evaluation uses an eligible research design.

## APPENDIX C FULL‐TEXT CODING FORM ^4^ This form has been informed by published coding forms (e.g., Littell, Corcoran, & Pillai, 2008; Higginson, Eggins &, Mazerolle, 2017; Mitchell, Wilson, Eggers, & MacKenzie, 2012). INCLUSION CRITERIA

General study details

1. Study ID [textbox]
2. Report ID [textbox]*
3. What type of document is this study? [*dropdown menu*]
  a. Peer‐reviewed journal article
  b. Book chapter
  c. Dissertation
  d. Conference presentation
  e. Government report, technical report, or working paper
  f. Other (specify in textbox)
4. In what country was the intervention implemented? [*textbox*]
5. If the evaluation and/or intervention was funded, record the funding source. [*textbox*]

*SysReview allows for multiple reports of a single study to be included in the one full‐text coding record form. Each report is nested within the overall study record and the Report ID will consist of the Study ID followed by a unique alphabetical code (e.g., 1234_a, 1234_b…).

Participants

1. Who are the participants? [*checkboxes*]
  a. Citizens
  b. Practitioners (specify in textbox)
  c. Micro places (specify in textbox)
  d. Macro places (specify in textbox)
  e. Other (specify in textbox)
2. How were participants recruited? [*textbox*]
3. What were the eligibility criteria for inclusion in the study? [*textbox*]
4. Describe sample attrition. [*textboxes*]
  **Table:**
  Number of participants	Treatment	Comparison	Total
  Referred to study			
  Consented			
  Assigned			
  Began intervention			
  Completed intervention			
  Completed follow‐up 1			
  Completed follow‐up 2 (if applicable)			
5. Describe the characteristics of the sample. [*textboxes*]
  **Table:**
  Number of participants	Treatment	Comparison	Total
  Age (M, SD, range)			
  Gender (% female)			
  Ethnicity (proportions)			
  Socioeconomic status (proportions)			
6. Record any other pertinent sample information for both the treatment and comparison groups. [*textbox*].

General methodological details and nature of comparisons

1. What is the nature of the comparisons for this study? [*dropdown menu*]
  a. Single intervention contrasted with single comparison condition
  b. Multiple interventions against a single comparison condition
  c. Within one group over time
  d. Other (specify in textbox)
2. General research design classification [*dropdown menu*]
  a. Randomised controlled trial
  b. Quasi‐randomised controlled trial
  c. Non‐randomised controlled trial (e.g., interrupted time‐series, matched control group design)
  d. Other (specify in textbox)
3. What type of comparison condition was used? [*dropdown menu*]
  a. No treatment
  b. Treatment‐as‐usual (specify in textbox)
  c. Alternative treatment (specify in textbox)
  d. Waitlist control
  e. Other (specify in textbox)
4. How were treatment and comparison groups formed? [*dropdown menu*]
  a. Random allocation
  b. Matching (specify matching method and matching variables in textbox)
  c. On basis of score on a specific measure (e.g., diagnosis, specify in textbox)
  d. Self‐selection
  e. Other (specify in textbox)
  f. Unclear
5. What was the unit of allocation? [*dropdown menu*]
  a. Participant
  b. Dyads
  c. Family
  d. Service site
  e. Other (specify in textbox)
  f. Unclear
6. If participants were randomly allocated to conditions, how was this implemented? [*dropdown menu*]
  a. Simple
  b. Yoked pairs
  c. Cluster (specify cluster in textbox)
  d. Block/stratified (specify variables in textbox)
  e. Matched pairs (specify matching variables in textbox)
  f. Other (specify in textbox)
  g. Unclear
  h. Not applicable
7. Who executed the randomisation? [*dropdown menu*]
  a. Researchers
  b. Practitioners
  c. Other (specify in textbox)
  d. Unclear
8. If applicable, was randomisation equivalent across intervention sites? [*dropdown menu*]
  a. Yes
  b. No
  c. Unclear
  d. No applicable
9. Was group equivalence assessed? [*dropdown menu*]
  a. Yes (specify how this was done in textbox)
  b. No
  c. Unclear
  d. Not applicable
10. Were the treatment and comparison groups equivalent at baseline? [*dropdown menu*]
  a. Yes
  b. No (specify differences)
  c. Unsure
  d. Not applicable
11. Are there any differences between participants who completed versus did not complete the treatment? [*dropdown menu*]
  a. Yes (specify differences)
  b. No
  c. Unsure
  d. Not applicable
12. What was the unit of analysis? [*dropdown menu*]
  a. Participant
  b. Dyads
  c. Family
  d. Service site
  e. Other (specify in textbox)

Unclear Intervention details

1. What is the name of the intervention(s), as reported by study authors? [*textbox*]
2. What settings were used during the intervention(s) (e.g., community, institutions etc)? [*textbox*]
3. When was the intervention conducted (e.g., year)? [*textbox*]
4. Describe the intervention provided to participants, ensuring you record the specific components or materials implemented and the mode of implementation. [*textbox*]
5. Describe the duration of the entire intervention. If reported, describe the minimum, maximum, mean and standard deviation for intervention duration. [*textbox*]
6. Describe the intensity of the intervention (e.g., frequency of contacts and length of contacts). If reported, describe the minimum, maximum, mean and standard deviation for intervention intensity. [*textbox*]
7. Who implemented the intervention? [*textbox*]
8. Was there more than one intervention site? [*dropdown menu*]
  a. Yes (specify number of sites in textbox)
  b. No
  c. Unclear
9. Was treatment integrity monitored? [*dropdown menu*]
  a. Yes (specify in textbox)
  b. No
  c. Unclear
10. Were there any issues with fidelity? [*dropdown menu*]
  a. Yes (specify in textbox)
  b. No
  c. Unclear
11. Did the authors report cost‐benefit data? [*dropdown menu*]
  a. Yes (specify in textbox)
  b. No
  c. Unclear

Outcome(s) measurement*

*To be completed for each eligible outcome within a study (or group of reports for a study). To add another outcome, click the “Add another outcome” button located at the bottom of the screen.

1. What is the outcome being measured? [*textbox*]
2. How was the outcome data gathered? [*dropdown menu*]
  a. Self‐report
  b. Observation
  c. Official source
  d. Interview
  e. Other (specify in textbox)
3. What are the psychometric properties of the measurement tool (e.g., reliability, validity, diagnostic thresholds, what higher /lower values mean)? [*textbox*]
4. Who was the respondent/participant? [*textbox*]
5. At what time‐point(s) was the outcome measured? [*textbox*]
6. Were data collected in the same manner for the treatment and comparison conditions? [*dropdown menu*]
  a. Yes
  b. No (specify in textbox)
  c. Unclear
7. Which condition does the raw difference/effect favour (ignore statistical significance)? [*dropdown menu*]
  a. Experimental condition
  b. Comparison condition
  c. Neither condition (no difference)
  d. Unclear
8. In which direction did the outcome change? [*dropdown menu*]
  a. Positive
  b. Negative
  c. Mixed (specify in textbox)
  d. Unclear
9. Were there statistically significant differences for this outcome? [*dropdown menu*]
  a. Yes
  b. No
  c. Not tested
  d. Unclear
10. What were the study author(s)' conclusions about this outcome? [*textbox*]

Effect size data*

*To be completed for each eligible outcome within a study (or group of reports for a study). To add another outcome, click the “Add another outcome” button located at the bottom of the screen.

1. On what page number is the effect size data reported? [*textbox*]
2. What type of effect size is being coded? [*dropdown menu*]
  a. Post‐intervention only (first point of measurement after intervention)
  b. Baseline and postintervention or pretest measure prior to intervention)
  c. Follow‐up (subsequent point of measurement after first post‐test)
3. What is the timeframe captured for the measure?
  a. Minimum [*textbox*]
  b. Maximum [*textbox*]
  c. Mean [*textbox*]
  d. Same for all participants (i.e., fixed) [*textbox*]
4. How was the effect size obtained for this outcome? [dropdown menu]
  a. Reported in document → Go to Question 5
  b. Calculated by user → Go to Question 6
5. Identify the type of effect size reported for this outcome and enter the required data for that effect size in the text boxes provided. [*textboxes*]
6. Enter the appropriate data in the relevant “Data for effect size calculations” tabs (see below). The data entered will depend on what is reported in the document. If none of the circumstances in the tabs reflect the data in the document, follow the link to David Wilson's online effect size calculator to calculate an effect size. You can enter the data in the “Data for effect size calculations 2” tab in the “Other information” textbox. [*textboxes*]
